# Iatrogenic Hepatic Pneumovenogram

**DOI:** 10.1155/2011/731758

**Published:** 2011-05-18

**Authors:** Govarthanan Rajendiran, Sulaiman Rathore, Gurmeet Sidhu, James Catevenis

**Affiliations:** Department of Critical Care Medicine, Prince George's Hospital Center, 3001 Hospital Drive, 5th Floor, Cheverly, MD, 20785, USA

## Abstract

Accidental air entry during central venous catheterization is a preventable iatrogenic complication that can cause venous air embolism (VAE). Many cases of VAE are subclinical with no adverse outcome and thus go unreported. Usually, when symptoms are present, they are nonspecific, and a high index of clinical suspicion of possible VAE is required to prompt investigations and initiate appropriate therapy. Occasionally large embolism can lead to life-threatening acute cor pulmonale, asystole, sudden death, and arterial air embolism in the presence of shunt or patent foramen ovale. This paper discusses VAE during emergency central line placement and the diagnostic dilemma that it can be created in critically ill patients. All necessary precautions have to be strictly followed to prevent this iatrogenic complication.

## 1. Introduction

Venous air embolism (VAE) is a subset of gas embolism which can result in serious morbidity and mortality. It is a common iatrogenic complication and most often associated with invasive vascular procedures, hemodialysis, central venous catheterization, high-pressure mechanical ventilation, thoracentesis, and diagnostic radiocontrast injection [1–5]. We report a case of VAE in a critically ill patient detected incidentally in CT imaging, and the air was found in the hepatic venous system. The major concerns were to exclude serious intra-abdominal pathology, such as bowel wall ischemia, intra-abdominal abscess, and septic thrombophlebitis that might have caused it. When such etiologies are excluded, it is essential to exactly pinpoint the iatrogenic cause, so that it can be prevented in the future. Most important is to prevent the entry of air into the systemic venous circulation that can result in acute cor pulmonale, reduced cardiac output, and cardiovascular collapse [6–8].

Generally small amount air in the venous system gets absorbed spontaneously without any sequelae, but rapid entry or large amount of air can result in significant morbidity and mortality. VAE has primary complications resulting from “air lock” in the right ventricular (RV) outflow tract increasing the RV strain and reducing the cardiac output.

## 2. Case Report

We present a 73-year-old female who was admitted for evaluation of syncope. Her past medical history included diabetes mellitus, hypertension, coronary artery disease, dementia, and schizophrenia. She had undergone coronary artery bypass graft surgery, cholecystectomy, and cervical spine surgery many years ago. She did not undergo any recent surgery or sustain trauma or head injury. Her examination findings were within normal limits. Laboratory findings were significant for mild anemia, mild prerenal insufficiency, and evidence of urinary infection. CT imaging of the brain, electroencephalogram, carotid doppler study, and chest X-ray were within normal limits. Her mild prerenal insufficiency improved with intravenous fluid hydration.

**Figure 1:**
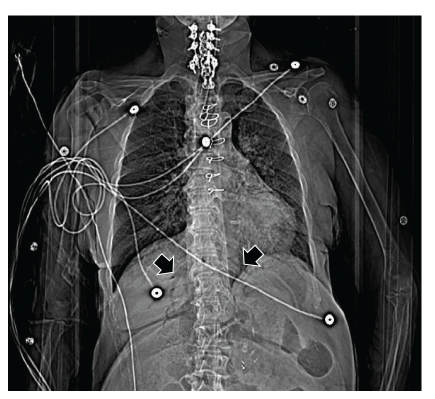
Scout film delineating the hepatic venous system secondary to air contrast (*arrows*).

On the next day of admission, she developed altered mental status and desaturation. She was hypoxic (SpO_2_ 82% on 100% FiO_2_) and hypoglycemic (blood glucose 37 mg/dL), but, despite correction of glucose level with intramuscular glucagon, she remained minimally responsive and hypoxia persisted. As her peripheral venous line was infiltrated, immediate bedside femoral central venous line was placed and peripheral line was removed. She was intubated for airway protection and acute respiratory failure. Immediately after central line placement, she underwent stat CT imaging of the chest with intravenous contrast medium (Pulmonary Embolism PE protocol) which did not show any evidence of pulmonary embolism. The scout film showed abnormal venogram involving air contrast in the hepatic venous system flowing towards inferior vena cava, and no air was seen in the portal venous system or biliary tract (Figure 1).

**Figure 2:**
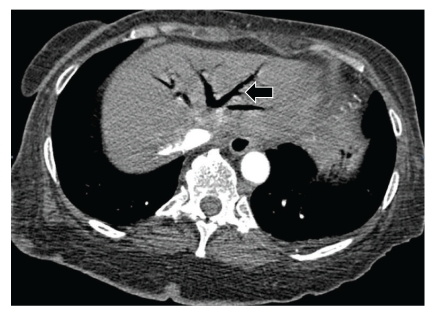
CT image of the liver shows air delineating the hepatic venous system (*arrow*).

**Figure 3:**
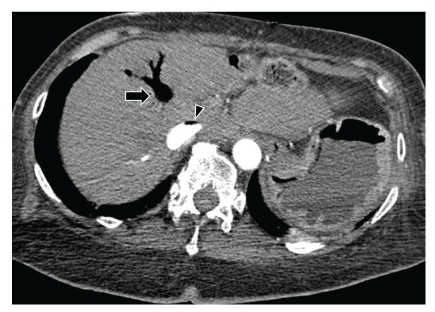
CT image of the liver shows air in the hepatic veins (*arrow*) and air-contrast level in the inferior vena cava (*arrowhead*).

Sequential images from lower sections of CT of the chest study showed air in the entire hepatic venous system and air-contrast level in the inferior vena cava (Figures 2 and 3). Bedside echocardiography did not show any evidence of air in the right atrium, right ventricle or pulmonary artery. Patient was ventilated with 100% FiO_2_ and positioned in the left lateral position.

**Figure 4:**
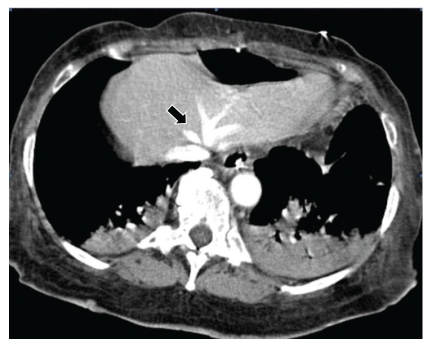
CT image of the liver shows normal contrast enhancement of hepatic venous system (*arrow*). It also shows bibasilar atelectasis.

**Figure 5:**
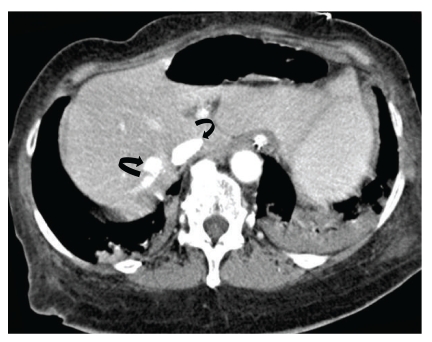
CT image of the liver shows normal contrast enhancement in the inferior vena cava and hepatic vein (*curved arrows*).

CT imaging of the abdomen with oral (via nasogastric tube) and intravenous contrast medium was performed to rule out intra-abdominal pathology. Normal contrast filled the hepatic venous system and inferior vena cava, but the air in the hepatic veins and inferior vena cava has disappeared in 2 hours (Figures 4 and 5). There was no evidence of bile duct stone or dilatation. Her gall bladder was absent secondary to cholecystectomy, and there was no evidence of bowel wall ischemia, inflammatory process, pancreatitis, or abscess.

## 3. Discussion

In this case, the air in the hepatic venous system was iatrogenic, and it probably happened with inadvertent air entry during central venous catheterization. VAE is a well-known complication of central venous placement [1, 2, 9] and can also happen during improper removal of the central venous line [10, 11].

Our major concern was to prevent embolism to right heart and pulmonary artery which was accomplished by high supplemental oxygen and patient positioning. Bedside echocardiography confirmed the absence of air bubbles in the right heart and pulmonary artery and did not show any evidence of right ventricular strain or dilatation. 

The next concern was to exclude the presence of serious abdominal conditions or sepsis in view of her clinical deterioration. In a critically ill patient, serious abdominal conditions that can lead to hepatic-portal venous gas have to be excluded before attributing it to iatrogenic complication, and they are summarized in Table 1 [7, 8]. Contrast-enhanced abdominal imaging helped us rule out most of the serious conditions.

It was necessary to differentiate the venous air pattern in the liver from pneumobilia, which is found centrally, that is, more than 2 cm away from the liver capsule due to the centripetal flow of bile [12, 13]. In this case the air column in the hepatic venous system was communicating with air level in the inferior vena cava, and there was no evidence of common bile duct air or obstruction. 

Equally important was to rule out accidental air injection along with radiocontrast injection, and this iatrogenic complication has been reported in the literature [3–5]. The differentiating feature was the presence of pneumovenogram in the scout film which implied that air embolism happened even before contrast injection. Our patient did neither have any evidence of serious abdominal pathology nor pneumobilia, and the air embolism happened before radiocontrast injection, which narrowed the etiology on inadvertent air entry during central venous catheterization. Air is easily entrained into the vascular space when a needle or catheter is left open to the atmosphere. Fatal doses of air measuring as little as 20 mL can be aspirated in seconds through a large bore catheter [8]. Upright positioning, hypovolemia, spontaneous inhalation during instrumentation, and inattention to catheter seals increase the risk for entraining air. Trendelenburg positioning, Valsalva maneuver, prompt needle/catheter occlusion, and tight intravenous connections help to avoid this complication [8]. 

Improper removal of central venous lines can also result in venous air embolism. Therefore, central venous catheters should be removed while the patient is either supine or in Trendelenburg position, and the insertion site must immediately be covered with a sterile gauze, with firm manual pressure maintained until hemostasis is achieved. The insertion site must then be covered with an air-occlusive dressing, and it should remain in place for 24 to 72 hours [10]. It is also important to remember that urgent care is required to prevent air embolism after the accidental removal of a central venous catheter. As our patient did not have any ongoing disease to maintain air accumulation, the air disappeared without causing any serious clinical consequences.

Rapid entry or large volume of air (over 50 mL) can lead to acute cor pulmonale, asystole, or both [6]. Obstruction of the pulmonary outflow tract “air lock” diminishes blood flow from the right heart and results in increased central venous pressure and reductions in pulmonary and systemic arterial pressures [7]. Smaller bubbles within the pulmonary arterioles can impede blood flow directly and result in vasoconstriction and also can result in secondary tissue damage from the release of inflammatory mediators and oxygen free radicals, as summarized in Table 2 [8]. Depending on the severity of air embolism, treatment includes high-flow supplemental oxygen, hyperbaric oxygen therapy, patient positioning (left lateral decubitus and Trendelenburg positions), closed chest cardiac massage, and intracardiac aspiration of air [8].

## 4. Conclusion

The educational objective of this paper is to stress the importance of strict precautions that need to be followed to prevent venous air entry during central venous catheterization, as well as with removal of central venous lines. Following safety precautions can prevent VAE and also will avoid unnecessary investigations and complications. Prompt management and improved outcome requires a high index of suspicion and early detection of venous air emboli.

## Figures and Tables

**Table 1:** Important causes of hepatic portal venous gas in critically ill patients.

Bowel wall ischemia
Intra-abdominal abscess
Septic thrombophlebitis
Suppurative cholangitis
Diverticulitis
Pancreatitis
Necrotizing enterocolitis
Inflammatory bowel disease
Intestinal obstruction
Paralytic ileus
Abdominal trauma (penetrating or blunt)

**Table 2:** Complications of venous air embolism.

Hemodynamic	Increased pulmonary vascular resistance
	Pulmonary artery hypertension
	Increased right ventricular pressure
	Decrease in cardiac output
	Myocardial ischemia

Pulmonary	Hypoxemia (from alveolar flooding and ventilation-perfusion mismatching)
	Increased physiologic dead space
	Decreased lung compliance secondary to pulmonary edema
	Increased airway resistance (postulated to be due to release of bronchoconstricting mediators such as serotonin and histamine from endothelium damaged by the air bubbles)

Systemic	Ischemic damage from microcirculation by air bubbles
	Secondary tissue damage from the release of inflammatory mediators and oxygen free radicals
